# The Effect of Betel-nut Chewing on the Buccal Mucosa of 296 Indians and Malays in West Malaysia. A Clinical Study

**DOI:** 10.1038/bjc.1970.50

**Published:** 1970-09

**Authors:** C. T. Chin, K. W. Lee

## Abstract

Changes in the buccal mucosa of 296 Indian and Malay betel-nut chewers in Perak, West Malaysia, were studied clinically. 167 out of 212 Indian subjects incorporated tobacco in their quids, while 45 out of 84 Malay subjects incorporated. “Gambir”. The Indians appeared to show a higher proportion of mucosal changes, particularly when tobacco was used. “Gambir” did not appear to be potent in the production of mucosal changes. Comparison with studies in other parts of the world suggested comparable findings with respect to both tobacco and non-tobacco chewing samples, and there would appear to be some evidence that tobacco-containing quids are likely to produce a higher proportion of mucosal changes as compared to non-tobacco-containing quids. An attempt to demonstrate a dose-effect relationship by dividing the subjects into “slight” and “heavy” chewers did not yield significant differences between these two categories in each of the groups.


### THE EFFECTS OF BETEL-NUT CHEWING ON THE BUCCAL MUCOSA OF 296 INDIANS AND MALAYS IN WEST MALAYSIA. A CLINICAL STUDY

C. T. CHIN* AND K. W. LEE

*From the Government Dental Clinic, Parit, Perak, West Malaysia, and the Department of Pathology, Institute of Dental Surgery, London*

Received for publication May 26, 1970

**SUMMARY.—Changes in the buccal mucosa of 296 Indian and Malay betel-nut chewers in Perak, West Malaysia, were studied clinically. 167 out of 212 Indian subjects incorporated tobacco in their quids, while 45 out of 84 Malay subjects incorporated " Gambir ". The Indians appeared to show a higher proportion of mucosal changes, particularly when tobacco was used. " Gambir " did not appear to be potent in the production of mucosal changes. Comparison with studies in other parts of the world suggested comparable findings with respect to both tobacco and non-tobacco chewing samples, and there would appear to be some evidence that tobacco-containing quids are likely to produce a higher proportion of mucosal changes as compared to non-tobacco-containing quids. An attempt to demonstrate a dose-effect relationship by dividing the subjects into " slight " and " heavy " chewers did not yield significant differences between these two categories in each of the groups.**

THE high incidence of oral cancer in South-East Asia has for long been linked with the habit of betel-nut chewing, particularly when tobacco has been incorporated into the quid. Hirayama (1966) in an extensive epidemiological study of oral and pharyngeal cancer in Central and South-East Asia showed (1) that there is a relationship between the site of cancer within the mouth and the site where the tobacco-containing quids are kept, (2) that there is a dose-response relationship and that the relative risk of developing a cancer of the mouth increases with the amount chewed, and (3) that within the mouth this relative risk for tobacco chewers is greater for the anterior parts of the mouth and not so great for the posterior parts. He concluded that the chewing of tobacco and lime mixtures play an important role in the aetiology of oral cancer in most parts of South-East Asia and Central Asia causing cancer at the place in which the quid is habitually put. He pointed out, however, that whether it was the tobacco or the lime which played the major role was a matter for future study, as it is also known that oral cancer is rare in territories where people chew tobacco without lime, and that it can be high in territories where betel-nut and lime are chewed without tobacco. The composition, chemistry and pharmacology of the quid have been reviewed by Muir and Kirk (1960).

The changes that occur in the oral mucosa as a result of betel-nut chewing in

* Present address: Dental Clinic, District Hospital, Kluang, Johore, West Malaysia.

individuals who have not, or not yet developed carcinomas, and the incidence of such changes have been studied in different parts of the world where the habit is wide-spread, with different results, a reflection perhaps of the differences in quid composition and the difficulty of categorizing the changes seen.

Most observers agree that the changes seen range from a roughness of the oral mucosa to the formation of a white patch which may be plaque-like, fissured and interspersed or bordered by erythematous zones. The term " leukoplakia " has been used to describe the changes seen without qualification as to its meaning. Waldron and Shafer (1960) have discussed the confusion that has accompanied the use of the term, and Silverman, Renstrup and Pindborg (1963) have defined " leukoplakia " as " a well-demarcated elevated white patch of 5 mm. or more in diameter which could not be scraped off and could not be attributed to the presence of other diseases ", in an attempt to standardize the nomenclature to allow comparative studies to be more easily made. Clinically discernible mucosal changes which do not meet this qualification, however, appear more difficult to categorize. Pindborg, Barmes and Roed-Peterson (1968) use the term "preleukoplakia" to denote " a lesion of the oral mucosa too vague in colour to be called manifest leukoplakia. The affected area usually presented a whitish or greyish hue and occasionally was slightly raised and could not be scraped off."

Oral cancer associated with betel-nut chewing is the only common form of oral cancer in Malaya and its frequency is particularly high in the Indian population (Marsden, 1960; Hirayama, 1966). However, the habit of betel-nut chewing is not restricted to the Indian population, for the Malays also chew betel, and yet seldom develop these cancers. Marsden points out that the Indians always include tobacco in their quids whilst Malays do not, and that this accounts for the difference.

It seemed, therefore, that it would be of interest to study the effects of betel-nut chewing on the oral mucosa in a multi-racial population such as exists in Malaysia, to see if differences can be found in different ethnic groups using different quids, and to see if a dose-effect relationship can be demonstrated in individuals before oral cancer has supervened.

### MATERIAL AND METHOD

One of us (C.T.C.) carried out a clinical examination of 296 subjects in two towns in Northern Malaya. The subjects consisted of known betel-nut chewers; 212 were Indians, 84 were Malays. All the Indian subjects were workers in two rubber estates in the neighbourhoods of Sitiawan and Parit, two towns 40 miles apart in the state of Perak. The Malay subjects were patients who presented for routine dental treatment at the Government Dental Clinic, Parit.

The following details of the habit were recorded: (a) Total duration of the habit. (b) Type of quid used. (c) Sites where quids were placed. (d) Frequency of chewing. (e) Duration of each chew. It had not been possible to enter into the smoking or alcohol habits of the subjects in the present series owing to factors beyond our control.

A clinical examination of the oral cavity was then carried out. Mucosal changes which conformed to the definition of " leukoplakia " as defined by Silverman *et al.* (1963) were recorded as such, while all other observable changes of the oral mucosa attributable to the habit were recorded as " preleukoplakia ".

*Differences in composition of the quid*

Pindborg, Kiaer, Gupta and Chawla (1967) reported 38 different habitual ways in which tobacco and/or betel-nut were used, and it is necessary therefore to categorize the method of usage, before comparative studies can be made. All subjects in the present series chewed betel-nut in the form of a quid or " pan ". The quid used by 167 Indian subjects consisted of a young betel-leaf (*Piper betle* L.), slaked stone lime, tobacco, and powdered or sliced dried betel-nut. Forty-five Indians used the quid without tobacco. The quid used by 45 Malay subjects consisted of a more mature betel-leaf of the same species, " getah gambir ", slaked stone lime, and fresh betel-nut, and 39 Malay subjects were non-gambir chewers. " Getah gambir " is an extract from the shrub *Uncaria gambir* containing catechin. A little bran is usually added, and the bran-catechin mixture is made into cakes (Muir and Kirk, 1960).

*Clinical data*

The age range of the 167 Indians who incorporated tobacco in their quids was from 19 to 74 years (mean 45·8 years) and their durations of the habit ranged from 2 months to 56 years (mean 21·2 years). The age range of the 45 non-tobacco chewing Indians was from 12 to 71 years (mean 41·8 years) and their durations of the habit ranged from one month to 45 years (mean 8·7 years). The ages of the 45 gambir chewing Malays ranged from 25 to 90 years (mean 52·2 years) and their durations of habit ranged from 3 months to 70 years (mean 20·4 years) while the ages of the 39 non-gambir chewing Malays ranged from 25 to 80 years (mean 43·9 years) and their durations of habit ranged from 9 months to 50 years (mean 11·5 years).

### RESULTS

One hundred and five (62·8%) tobacco chewing Indians exhibited clinical discernible changes of the buccal mucosa. Sixty-seven (40·1%) were designated " leukoplakia " and 38 (22·7%) were designated " preleukoplakia ". Twenty-one non-tobacco chewing Indians (46·6%) showed discernible changes, 9 (20%) were " leukoplakias " and 12 (26·6%) were " preleukoplakias ". Ten gambir chewing Malays (22·2%) showed discernible changes (5 (11·1%) each for " leukoplakia " and " preleukoplakia "), 10 non-gambir chewing Malays (25·6%) showed discernible changes (5 (12·8%) each for " leukoplakia " and " preleukoplakia "). The results are summarized in Table I. To make comparisons possible with Atkinson *et al.* (1964) and Pindborg *et al.*'s (1968) studies in New Guineans the results are presented in similar form to their studies in Table II.

The Indians would appear to show a higher proportion of changes as compared to the Malays, particularly when tobacco has been incorporated into the quid. Gambir does not appear to be particularly potent in the production of mucosal changes. However, our samples are probably insufficiently homogenous to allow valid comparisons to be made.

An attempt was made to ascertain if a dose-effect relationship could be demonstrated in our sample of subjects. Hirayama (1966) has pointed out that the " heaviness " of the habit is difficult to define, and it is necessary to take into consideration the total duration of the habit, the frequency of chewing and the duration per chew. Because of the number of variables involved, the subjects

TABLE I.—*Prevalences of Preleukoplakia and Leukoplakia among 296 Indians and Malays*

| Race | Age-groups <30 M | <30 F | 30–50 M | 30–50 F | >50 M | >50 F | Total number examined | Pre-leuko-plakia No. | Pre-leuko-plakia % | Leuko-plakia No. | Leuko-plakia % | Both changes combined No. | Both changes combined % |
|---|---|---|---|---|---|---|---|---|---|---|---|---|---|
| *Indian* | | | | | | | | | | | | | |
| Tobacco | 2 | 7 | 37 | 53 | 29 | 39 | 167 | 38 | 22·7 | 67 | 40·1 | 105 | 62·8 |
| Non-tobacco | 1 | 6 | 3 | 24 | 4 | 7 | 45 | 12 | 26·6 | 9 | 20·0 | 21 | 46·6 |
| *Malay* | | | | | | | | | | | | | |
| Gambir | 1 | 1 | 3 | 16 | 6 | 18 | 45 | 5 | 11·1 | 5 | 11·1 | 10 | 22·2 |
| Non-gambir | 0 | 4 | 2 | 20 | 4 | 9 | 39 | 5 | 12·8 | 5 | 12·8 | 10 | 25·6 |
| Total | 4 | 18 | 45 | 113 | 43 | 73 | 296 | 60 | 20·2 | 86 | 29·1 | 146 | 49·3 |

TABLE II.—*Distribution of Study Sample According to Race, Sex, Type of Lesion and Category*

| Race | Diagnoses and habits | Males No. | Males % | Females No. | Females % | Total No. | Total % |
|---|---|---|---|---|---|---|---|
| Indian | Total examined | 76 | 100 | 136 | 100 | 212 | 100 |
| | Preleukoplakia | 18 | 23·7 | 32 | 23·5 | 50 | 23·6 |
| | Leukoplakia | 25 | 32·9 | 51 | 37·5 | 76 | 35·8 |
| | Tobacco | 68 | 89·5 | 99 | 72·8 | 167 | 78·8 |
| | Non-tobacco | 8 | 10·5 | 37 | 27·2 | 45 | 21·2 |
| Malay | Total examined | 16 | 100 | 68 | 100 | 84 | 100 |
| | Preleukoplakia | 0 | 0 | 10 | 14·7 | 10 | 11·9 |
| | Leukoplakia | 4 | 25 | 6 | 8·8 | 10 | 11·9 |
| | Gambir | 10 | 62·5 | 35 | 51·5 | 45 | 53·5 |
| | Non-gambir | 6 | 37·5 | 33 | 48·5 | 39 | 46·4 |

in the four groups were arbitrarily divided into two sub-groups. Those who gave a history of chewing for less than 10 years and whose " intensity of habit " (Meyer, Daftary and Pindborg, 1967), *i.e.* the product of the number of quids per day and the duration of each chew, was less than one hour, were categorized as " slight chewers ". Those who gave a history of chewing for more than 10 years or whose " intensity of habit " was more than one hour per day were categorized as " heavy chewers ". The proportion of subjects who exhibited discernible changes in the buccal mucosa was compared to those who did not in each of the four categories (Table III). The $X^2$ test failed to show any significant difference between the two categories in each of the groups, and it was concluded that on the basis of the criteria chosen, a dose-effect relationship could not be demonstrated in the present series.

### DISCUSSION

The heterogenous nature of our sample and the presence of single examiner bias make valid comparisons with other studies difficult. However, it is of interest to note that Chang (1966) observed discernible clinical changes in the oral mucosa in 56 out of 174 betel-nut chewers (32·1%) in Taiwan who do not use tobacco but use a shell-lime and betel-nut mixture. Pindborg, Barmes and Roed-Peterson (1968) studied the prevalence of leukoedema, preleukoplakia and leukoplakia among Papuans and New Guineans. In the North Coast Area where 95% of the study sample examined were betel-nut chewers (non-tobacco) 62 out of 283

TABLE III.—*Distribution of Study Sample According to Race, Category and "Heaviness" of Habit*

| Race | Slight chewers | | Heavy chewers | |
|---|---|---|---|---|
| | No. examined | No. exhibiting changes | No. examined | No. exhibiting changes |
| Indian | | | | |
| Tobacco | 37 | 20 (56%) | 130 | 85 (65%) |
| Non-tobacco | 29 | 11 (38%) | 16 | 10 (62%) |
| Malay | | | | |
| Gambir | 17 | 3 (17%) | 28 | 7 (25%) |
| Non-gambir | 17 | 1 (6%) | 22 | 9 (41%) |

Slight chewers = Less than 10 years' history of habit and "intensity of habit" less than one hour per day.

Heavy chewers = More than 10 years' history of habit or "intensity of habit" greater than one hour per day.

(19·9%) subjects exhibited either leukoedema (4·2%) preleukoplakia (12·0%) or leukoplakia (5·0%). Ahluwalia and Ponnampalam (1968) examined 168 Indian betel-nut chewers in Kuala Lumpur, Malaysia. Ninety-seven were females and, of these, 47 used tobacco-containing quids and 50 were non-tobacco chewers. The proportions of those who exhibited discernible changes in those two groups were 35 (74·4%) and 13 (26%) respectively. Seventy-one were men and, of these, 40 showed lesions (34 chewed tobacco and 6 were non-tobacco chewers).

In Pindborg *et al.*'s (1967) study of 10,000 individuals in Lucknow, India, however, only 40 out of 798 individuals (5·3%) who chewed tobacco-containing quids alone or in association with other tobacco habits exhibited leukoplakia. Of the individuals who chewed non-tobacco-containing quids only 6 out of 181 (3·3%) exhibited leukoplakia.

The combined figures for the three non-tobacco-containing groups in the present series show that 41 out of 129 subjects (31·7%) exhibit a discernible change in the buccal mucosa, with a range of 22·2–46·6%. The percentage figures would correlate well with the Taiwan study and Pindborg *et al.*'s (1968) New Guinea study, but would appear much higher than their Lucknow study. With respect to the tobacco chewing group, the present figures would be comparable to Ahluwahlia and Ponnampalam's (1968) study in Kuala Lumpur, but would appear high as compared to Pindborg *et al.*'s (1967) study in Lucknow. Thus, there would appear to be some evidence to suggest that tobacco-containing quids are likely to produce a higher proportion of mucosal changes as compared to non-tobacco-containing quids. Gambir, although suspect as a carcinogen or co-carcinogen (Korp'assy and Mosonyi, 1950; Kirby, 1960, quoted by Dunham *et al.*, 1966) would appear to be relatively ineffective in the production of mucosal changes.

It has not been possible to exclude the factor of smoking in the production of the observed mucosal changes. The subjects investigated come from a low socio-economic group and as Muir and Kirk (1960) point out betel chewing is a poor man's luxury, a made-up quid costing about the price of a cigarette but lasting much longer. The lesions observed bore a constant relationship to the sites where the quids were habitually kept, so that it can at least be assumed that their appearance is closely associated with the habit. Ahluwalia and Ponnampalam (1968), however, found that lesions were not necessarily produced at the sites where the quids were kept, and in their sample 96% of their male subjects smoked cigarettes and took varying amounts of liquor of different brands while few of the females smoked or took strong drinks. No examples of frank nicotinic stomatitis have been included in the present series, and as the role of smoking in the production of these changes is somewhat equivocal, it seems unlikely that it is of primary importance.

In recent years, submucous fibrosis of the oral mucosa, a condition seen mainly among Indians, has become increasingly regarded as a precancerous lesion (Pindborg, 1966). No patient in the present series presented with marked fibrosis of the oral tissues, although coexistence of the condition in its initial stages cannot be excluded with certainty. The changes seen in the present study may therefore represent a combination of submucous fibrosis, smoking and betel-nut chewing, at least in some patients.

Hirayama (1966) has demonstrated that a dose-effect relationship exists between the tobacco chewing habit and oral cancer, with a steady increase of the risk of developing oral cancer as the frequency of chewing increased. The complex number of variables involved makes an assessment of the " heaviness " of the habit difficult, and the arbitrary criteria selected in the present study has proved inadequate to show any statistically significant differences in the incidence of mucosal changes between the " slight " and " heavy " chewers. More refined criteria will have to be evolved to test if a dose-effect relationship can be demonstrated in the oral mucosa of individuals who have not yet developed cancers.

We gratefully acknowledge the permission of the Director General of Medical Services, Malaysia, to publish the results of the present study. We would like to thank the Director of Dental Services, Malaysia, and the Principal Dental Officer, Perak, for their encouragement, and the staff, District Health Centre, Parit, Perak, for their help. We would also like to thank the Director of the Royal Botanical Gardens, Kew, Richmond, Surrey, for the identification of the betel-leaves used by the subjects in the present study, and Dr. J. T. Boyd of the Medical Research Council Statistical Research Unit for his valuable advice.

### REFERENCES

AHLUWALIA, H. S. AND PONNAMPALAM, J. T.—(1968) *J. trop.Med. Hyg.*, **71**, 48.
ATKINSON, L., CHESTER, I. C., SMYTH, F. G. AND TEN SEDLAM, R. E. J.—(1964) *Cancer, N.Y.*, **17**, 1289.
CHANG, K. M.—(1966) *J. Formosan med. Ass.*, **65**, 79.
DUNHAM, L. J., MUIR, C. S. AND HAMNER III, J. E.—(1966) *Br. J. Cancer*, **20**, 588.
HIRAYAMA, T.—(1966) *Bull. Wld Hlth Org.*, **34**, 41.
MARSDEN, A. T. H.—(1960) *Med. J. Malaya*, **14**, 162.
MEYER, J., DAFTARY, D. K. AND PINDBORG, J. J.—(1967) *Acta odont. scand.*, **25**, 397.
MUIR, C. S. AND KIRK, R.—(1960) *Br. J. Cancer*, **14**, 597.
PINDBORG, J. J.—(1966) *J. dent. Res.*, **45**, 546.
PINDBORG, J. J., BARMES, D. AND ROED-PETERSON, B.—(1968) *Cancer, N.Y.*, **22**, 379.
PINDBORG, J. J., KIAER, J., GUPTA, P. C. AND CHAWLA, T. N.—(1967) *Bull. Wld Hlth Org.*, **37**, 1.
SILVERMAN JR., S., RENSTRUP, G. AND PINDBORG, J. J.—(1963) *Acta odont. scand.*, **21**, 271.
WALDRON, C. A. AND SHAFER, W. G.—(1960) *Int. dent. J.*, **10**, 350.


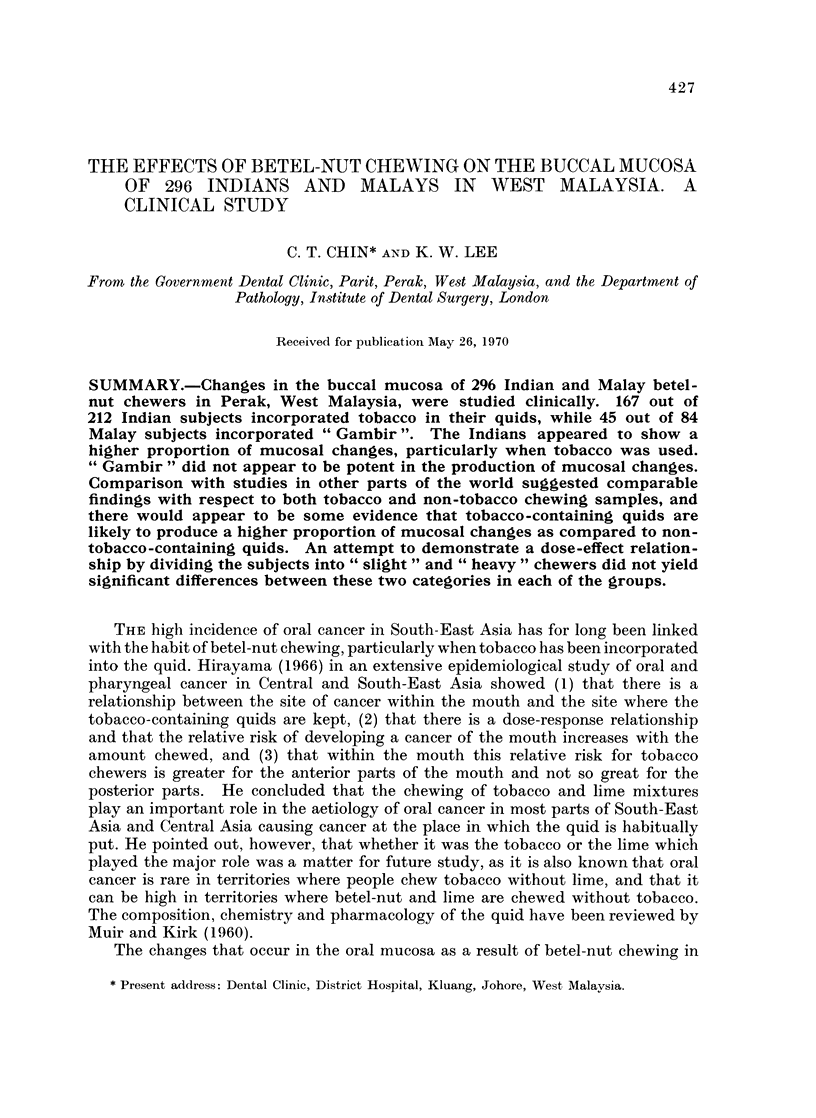

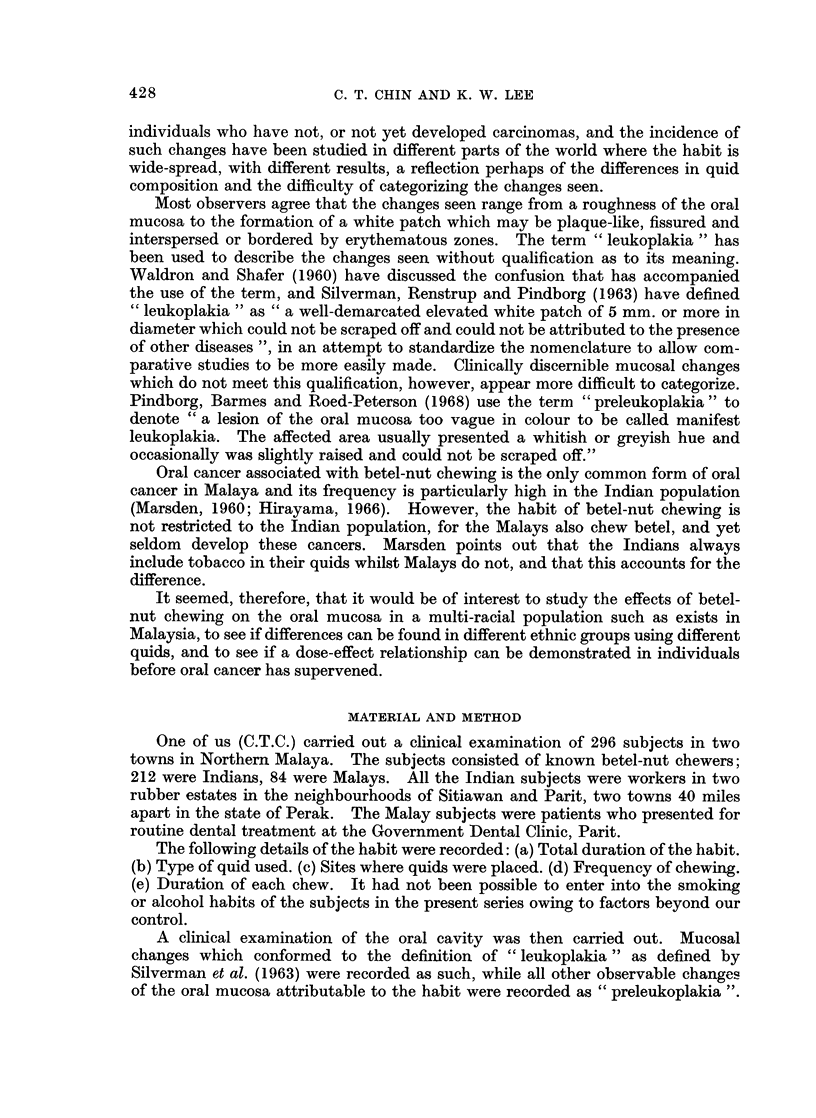

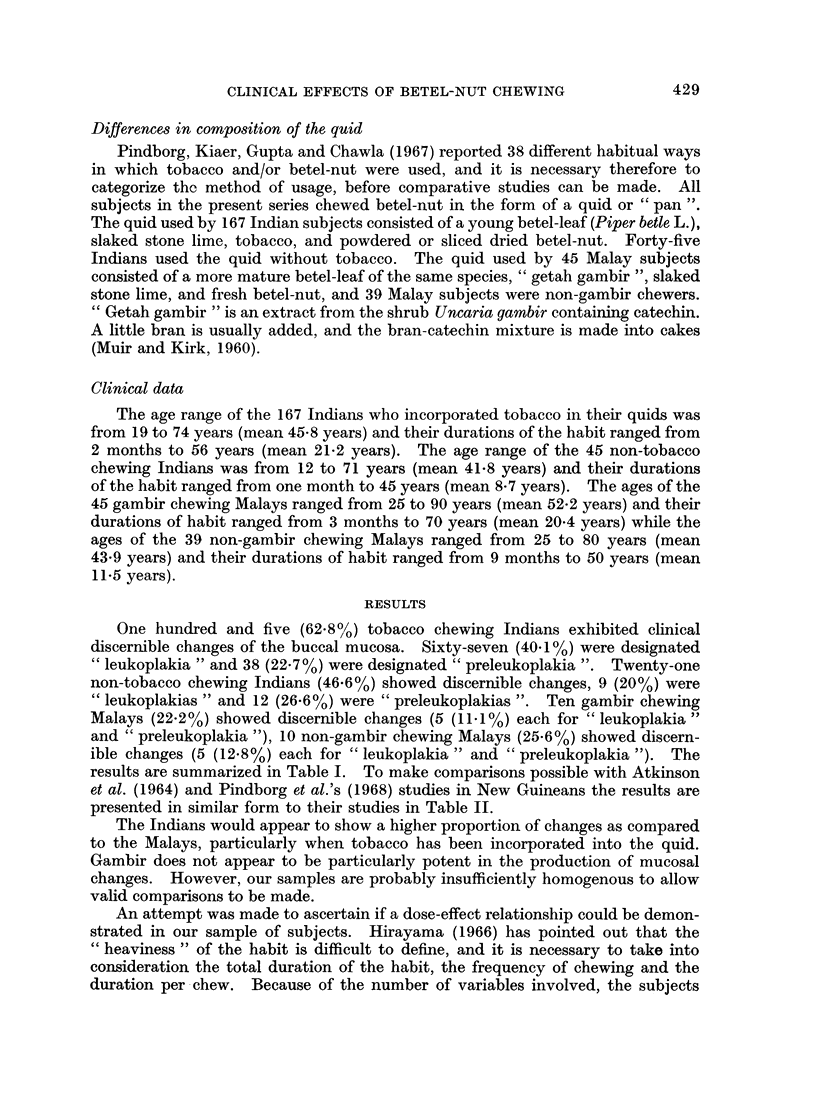

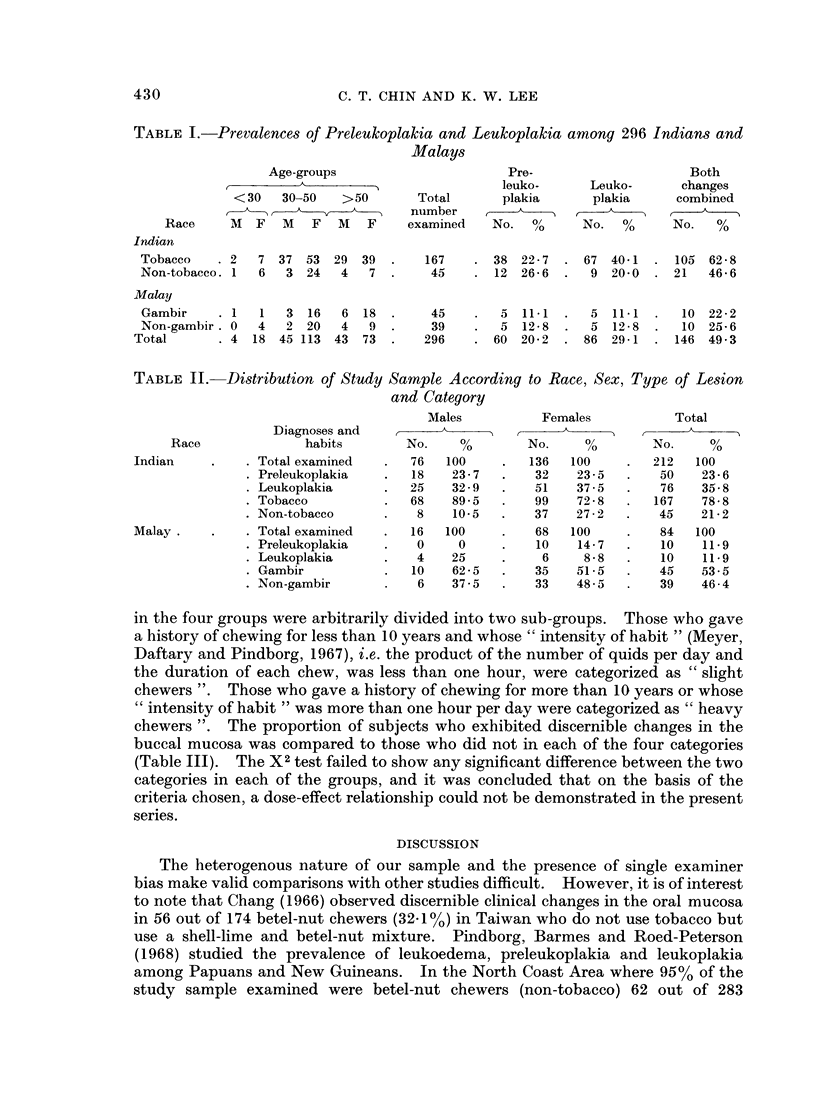

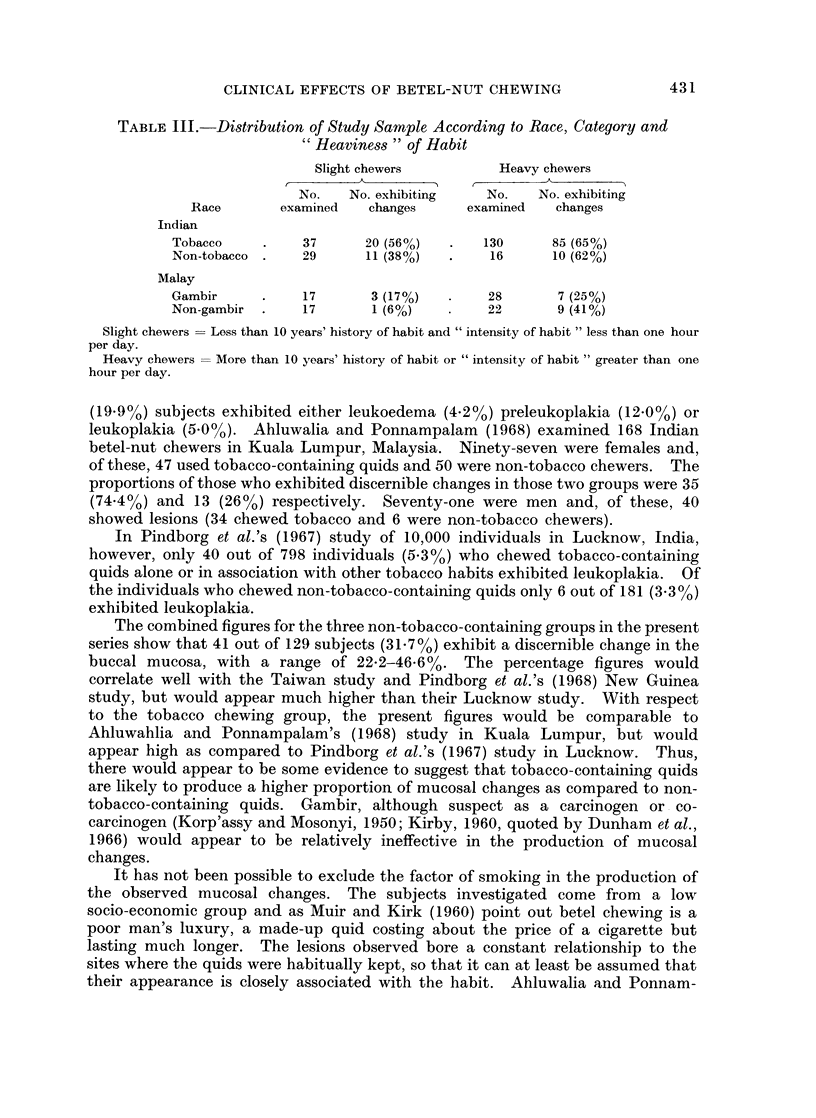

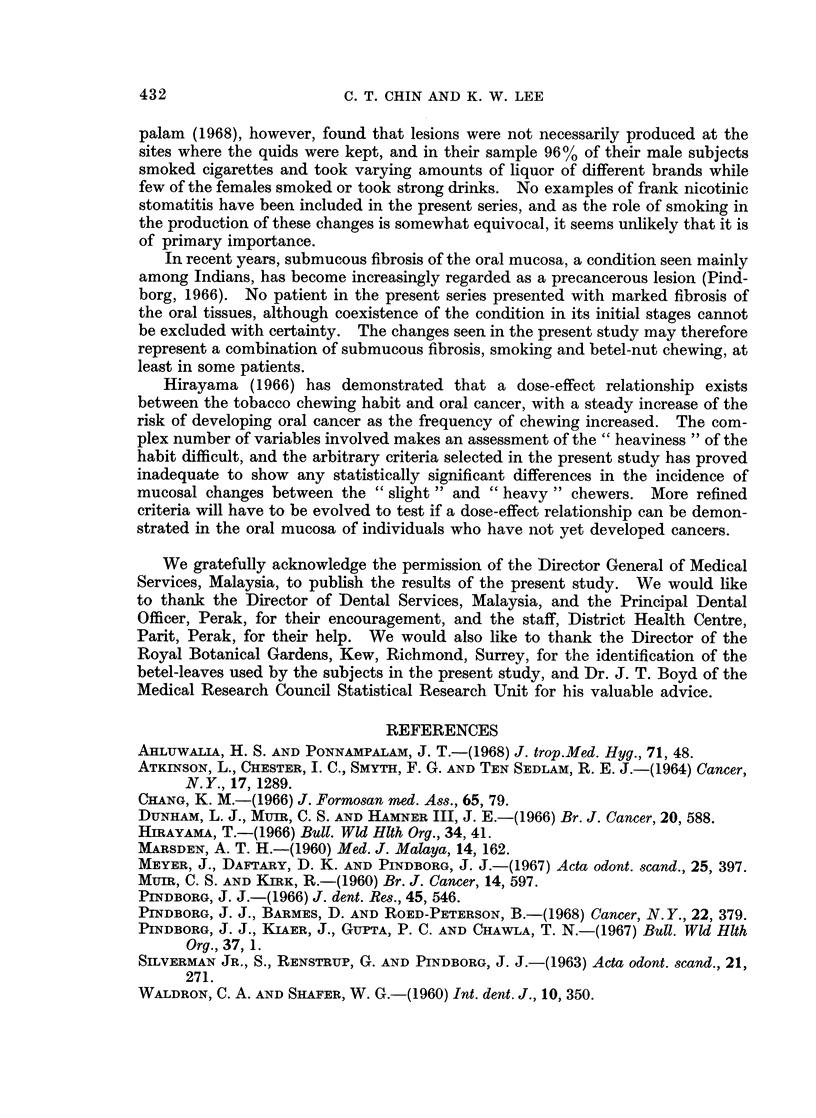


## References

[OCR_00349] ATKINSON L., CHESTER I. C., SMYTH F. G., TEN SELDAMR (1964). ORAL CANCER IN NEW GUINEA. A STUDY IN DEMOGRAPHY AND ETIOLOGY.. Cancer.

[OCR_00353] Dunham L. J., Muir C. S., Hamner J. E. (1966). Epithelial atypia in hamster cheek pouches treated repeatedly with calcium hydroxide.. Br J Cancer.

[OCR_00358] Meyer J., Daftary D. K., Pindborg J. J. (1967). Studies in oral leukoplakias. XI. Histopathology of leukoplakias in Indians chewing "pan" with tobacco.. Acta Odontol Scand.

[OCR_00362] Pindborg J. J., Barmes D., Roed-Petersen B. (1968). Epidemiology and histology of oral leukoplakia and leukoedema among Papuans and New Guineans.. Cancer.

[OCR_00371] WALDRON C. A., SHAFER W. G. (1960). Current concepts of leukoplakia.. Int Dent J.

